# Comprehensive analysis of juvenile idiopathic arthritis patients’ immune characteristics based on bulk and single-cell sequencing data

**DOI:** 10.3389/fmolb.2024.1359235

**Published:** 2024-05-01

**Authors:** Mubo Liu, Yadong Gong, Mu Lin, Qingqing Ma

**Affiliations:** Guizhou Aerospace Hospital, Zunyi, China

**Keywords:** juvenile idiopathic arthritis, single-cell RNA sequencing, immune repertoire, T-cell receptor, B-cell receptor

## Abstract

**Background:**

The pathogenesis of juvenile idiopathic arthritis (JIA) is strongly influenced by an impaired immune system. However, the molecular mechanisms underlying its development and progression have not been elucidated. In this study, the computational methods TRUST4 were used to construct a T-cell receptor (TCR) and B-cell receptor (BCR) repertoire from the peripheral blood of JIA patients *via* bulk RNA-seq data, after which the clonality and diversity of the immune repertoire were analyzed.

**Results:**

Our findings revealed significant differences in the frequency of clonotypes between the JIA and healthy control groups in terms of the TCR and BCR repertoires. This work identified specific V genes and J genes in TCRs and BCRs that could be used to expand our understanding of JIA. After single-cell RNA analysis, the relative percentages of CD14 monocytes were significantly greater in the JIA group. Cell-cell communication analysis revealed the significant role of the MIF signaling pathway in JIA.

**Conclusion:**

In conclusion, this work describes the immune features of both the TCR and BCR repertoires under JIA conditions and provides novel insight into immunotherapy for JIA.

## 1 Introduction

Juvenile idiopathic arthritis (JIA) is the predominant rheumatic disorder in children, encompassing various forms of persistent arthritis in childhood that affect joints and extra-articular structures, potentially resulting in disability or mortality (Zaripova et al., 2021; Ambler et al., 2022; Luo and Tang, 2024). As per the International League of Associations for Rheumatology, JIA is categorized into subtypes: oligoarticular, polyarticular, systemic, psoriatic arthritis, and enthesitis-related arthritis, each differing in genetic predisposition and severity of arthritis. The onset of the pathophysiological process in JIA involves aberrant activation of immunocytes, such as lymphocytes, natural killer cells, macrophages, and neutrophils, leading to the release of pro-inflammatory mediators that contribute to joint damage and systemic complications (Ling et al., 2012; Zhang W. et al., 2023).

A prior study indicates that the features of T-cell receptor (TCR) and B-cell receptor (BCR) repertoires could aid in comprehending adaptive immunity in autoimmune conditions (Attaf et al., 2015; Kim and Park, 2019). TRUST4, a highly efficient technique, can accurately infer TCR and BCR repertoires from bulk RNA-seq or single-cell sequencing (scRNA-seq) data. This approach has been shown to be effective in extracting immune profiles from bulk RNA-seq data without the need for additional T or B-cell receptor sequencing, thus reducing the cost of profiling immune repertoires in autoimmune diseases. Moreover, this approach has demonstrated exceptional efficiency, sensitivity, and precision in reconstructing CDR3s and can be used to assemble complete immune receptor sequences (Song et al., 2021; Liu and Han, 2022). The previous bioinformatics analysis results showed an increase in certain specific CDR3 amino acid sequences associated with autoimmune diseases, such as systemic lupus erythematosus and rheumatoid arthritis (Zhang and Lee, 2022). Hence, we hypothesize that certain specific CDR3 amino acid sequences also increased in JIA.

scRNA-seq is a cutting-edge technique that allows for the sequencing of DNA or RNA at the individual cell level (Ziegenhain et al., 2017; Slovin et al., 2021; Jovic et al., 2022). This approach provides a comprehensive understanding of the genetic architecture and gene expression patterns of individual cells, thereby greatly expanding our knowledge of transcriptional heterogeneity and dynamics across diverse biological entities.

In this study, we employed TCR and BCR repertoire analysis methods, along with integrated analysis *via* high-dimensional weighted gene coexpression network analysis (HdWGCNA) and cell trajectory tools, on bulk RNA-seq and scRNA-seq samples from healthy control (HC) and JIA patients. The aim was to characterize the changes in the transcriptional profile between the HC and JIA. The findings of this study offer novel insights into JIA treatment and provide fundamental research support for the clinical management of this disease.

## 2 Methods

### 2.1 Data sources

Peripheral blood mononuclear cells (PBMCs) of bulk RNA-seq data from the GEO datasets (https://www.ncbi.nlm.nih.gov/bioproject/?term=PRJNA439192). PBMC of scRNA-seq data from 6 JIA and 2 HC samples were obtained, specifically GSE205095 (https://www.ncbi.nlm.nih.gov/geo/query/acc.cgi?acc=GSE205095).

#### 2.1.1 T and B-cell receptor repertoire construction and analysis

To construct TCR and BCR repertoires, we employed the TRUST4. In brief: 1)Calculating the frequency of clonotypes, followed by Student’s t-test to compare JIA and HC groups. 2) Assessing TRBV, TRBJ, IGHV, and IGHJ gene proportions, with Student’s t-test identifying significantly altered genes. 3) Evaluating diversity in CDR3 amino acid sequences using InvSimpson and Chao1 indices. 4) Analyzing the distribution of CDR3 amino acid sequence lengths. 5) Examining the top 10 TCR and BCR V region motifs.

#### 2.1.2 Cell filtering and normalization

To mitigate interference from dead cells and cellular debris, this study adjusted for gene expression, and mitochondrial gene expression ratio in each cell sample. The threshold is as follows: cells with a total gene count >2,500, <200 genes, or a mitochondrial gene percentage <5% were excluded. The LogNormalize method is utilized to logarithmically transform and standardize gene expression values of cells, ensuring consistent total RNA expression levels across all cells.

#### 2.1.3 Data dimensionality reduction and UMAP analysis

Data were first normalized using the ScaleData function, which ensures that the data are on a comparable scale. Subsequently, we utilized the RunPCA function to reduce the dimensionality of the normalized data. To explore relationships between neighboring cells, the FindNeighbor function calculated distances based on a specified principal fraction (pcSelect) and identified nearest neighbors for each cell. The FindClusters function utilized the Leiden algorithm to assign cells to clusters based on the adjacency graph, with a resolution parameter of 2.0 for clustering. Lastly, the RunUMAP function computed UMAP coordinates, guided by the input principal fraction (pcSelect).

#### 2.1.4 Marker genes identification

To detect hypervariable genes, we performed variable gene selection for each sample, and a later integration analysis was performed to identify the 2,000 largest genes based on the overall gene mean and dispersion to conduct downstream analysis of the data for clustering, differential analysis, etc.

#### 2.1.5 Cell annotation

Cells were annotated using manual annotation, and the “FindAllMarkers” function was used to find markers for each cluster. The clusters were subsequently classified according to the characteristic gene expression of a specific cell type to annotate cells across multiple reference sets.

#### 2.1.6 HdWGCNA analysis

HdWGCNA utilized the R package ‘HdWGCNA’ to build a scale-free network. By establishing a scale-free topology model fit threshold of ≥0.80 and selecting a soft threshold of 9 for optimal connectivity, AUCell assessed the scRNA cohort using modules. The construction of a protein-protein interaction (PPI) was performed using the ‘HubGeneNetworkPlot’ function.

#### 2.1.7 Cell-cell communication analysis

For exploring cell-cell interaction, we employed CellChat (version 1.6.1) (Jin et al., 2021). In brief, we used the ‘createCellChat’ function to generate a CellChat object, allowing for the calculation of communication probabilities and the inference of cell interaction networks. Moreover, we delved into cell interaction at the signaling pathway level, allowing for the inference of inter-cellular interaction based on specific signaling pathway involvement.

#### 2.1.8 Trajectory analysis

For exploring trajectory analysis of cells, we employed the Monocle2 software package (version 2.28.0) (Zhang Q. et al., 2023). The UMI matrix was imported from the Seurat object, and the newCellDataSet function was employed to generate the object. Genes with a mean expression >0.1 were selected, followed by dimensionality reduction using the DDRTree method and cell ordering through the orderCells function.

#### 2.1.9 Transcriptomic factors analysis

For transcriptomic factors analysis, we employed pySCENIC (version 1.2.4). In summary, GENIE3 was employed to discover potential target genes of transcription factors, and the activity of each regulon within a cell was assessed by computing the area under the receiver operating characteristic curve using AUCell (version 1.16.0). Lastly, we used R script for cluster analysis and visualization of RegulonAUC data.

#### 2.1.10 Statistical analysis

Student’s t-test was performed to test for 1) the relative frequency of the 10 most abundant clonotypes between JIA and HC group 2) Chao1 and InvSimpson index of CDR3 amino acid sequence length between JIA and HC group; and 3) differentially expressed V genes and J genes between JIA and HC group. The Wilcoxon rank-sum test was employed, and a significance level of *p* < 0.05 was used to indicate a statistically significant difference. All statistical data were analyzed using R scripts.

## 3 Results

### 3.1 TCRs analysis

**FIGURE 1:**
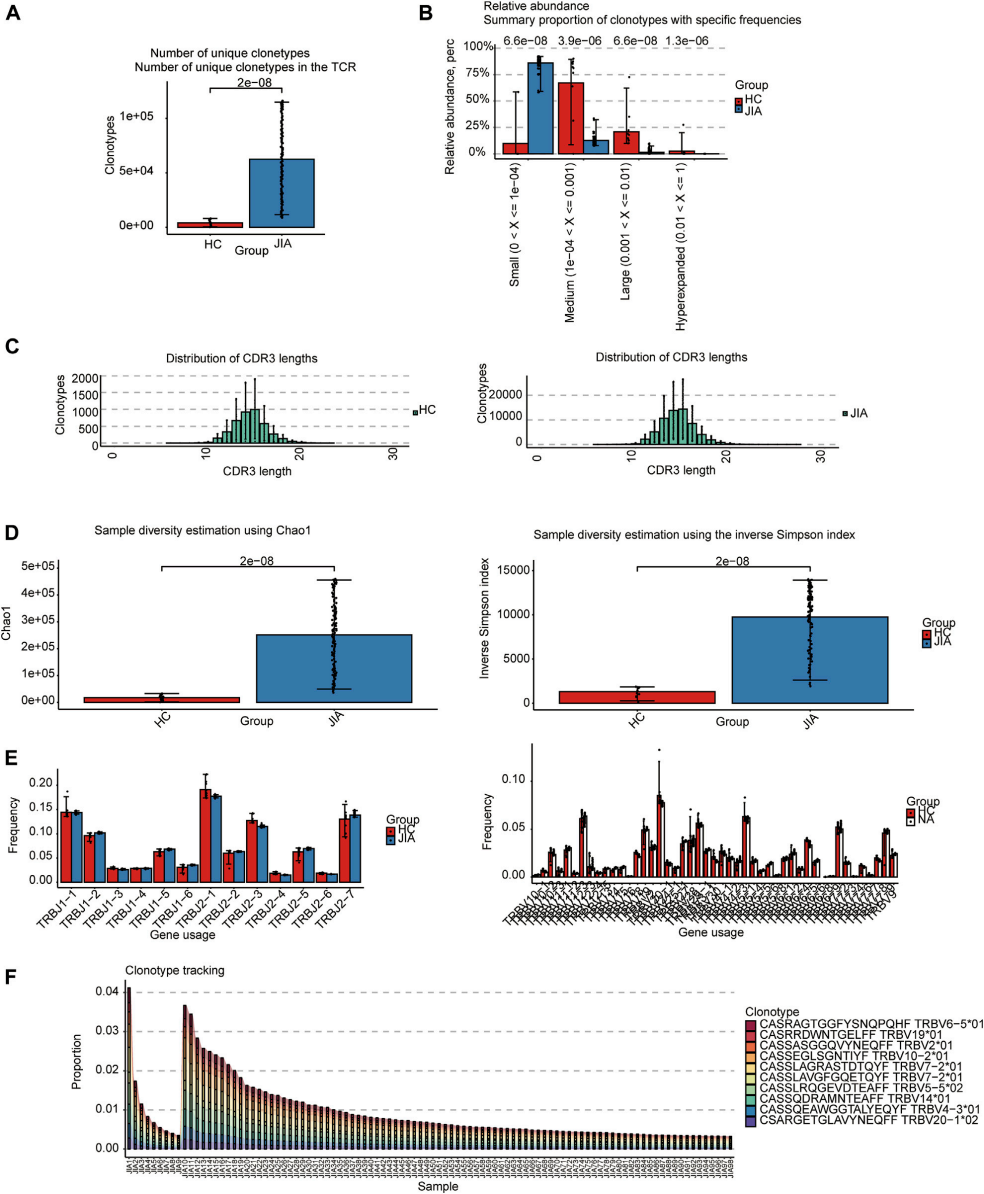
Characteristics of the JIA and HC immune repertoires (TCR repertoires). **(A)** Comparison of clonotype numbers for the TCR repertoire. **(B)** The relative abundance of TCR clonotypes with varying frequencies in the JIA and HC groups. **(C)** Distributions of TCR CDR3 amino acid sequence length in the JIA and HC groups. **(D)** Comparison of the differences in the clonotype diversity indicators Chao1 and InvSimpson indices. **(E)** Comparison of the Gene Usages of TRBV and TRBJ. **(F)** Clonotype tracking of JIA samples. Bar average, error bar standard error. *p* values were obtained by the Wilcoxon test.

The TCRs were analyzed in the bulk RNA-seq data using the standard workflow of TRUST4. The results showed that the frequency of clonotypes in the JIA group was significantly increased (*p* < 0.05; Figure 1A). Additional analyses were performed to examine the distribution of TCR clonotypes frequencies, uncovering categories of small, medium, large, and hyperexpanded frequencies. The clonotype distributions at small frequencies were significantly increased in JIA (*p* < 0.05; Figure 1B), whereas the distributions at medium and large frequencies and hyperexpanded frequencies were increased in HC(*p* < 0.05). The results suggested that the JIA exhibited a greater degree of dominant clonal expansion and a lower TCR repertoire diversity. The results of distributions of CDR3 amino acid sequence length showed that the most common CDR3 amino acid sequences for both JIA and HC are 15. CDR3 amino acid sequence length did not significantly differ between the JIA and HC groups (Figure 1C). Both the Chao1 and InvSimpson indices were utilized to evaluate the diversity of the CDR3 amino acid sequences. The JIA group exhibited significantly greater Chao1 and InvSimpson indices than the HC(*p* < 0.05; Figure 1D). To measure the frequency of TRBJ and TRBV genes, we generated a bar chart depicting the common usage frequencies of these genes (Figures 1E,F). The results showed that there was no difference between these two groups.

#### 3.1.1 BCRs analysis

**FIGURE 2:**
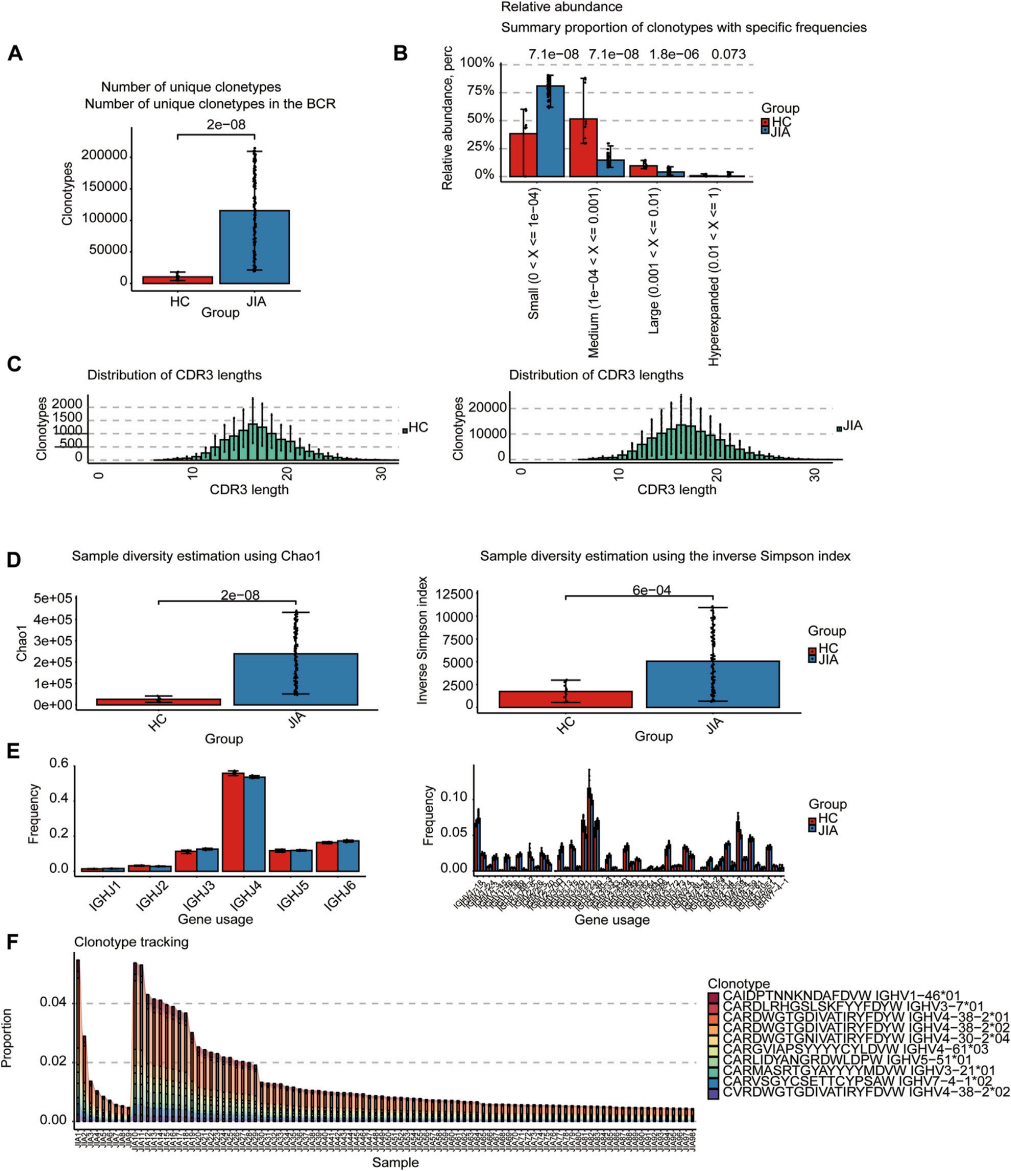
Characteristics of the JIA and HC immune repertoires (BCR repertoires). **(A)** Comparison of clonotype numbers for the BCR repertoire. **(B)** The relative abundance of BCR clonotypes with varying frequencies in the JIA and HC groups. **(C)** Distributions of BCR CDR3 amino acid sequence length in the JIA and HC groups. **(D)** Comparison of the differences in the clonotype diversity indicators Chao1 and InvSimpson indices. **(E)** Comparison of the Gene Usages of TRBV and TRBJ. **(F)** Clonotype tracking of JIA samples. Bar average, error bar standard error. *p* values were obtained by the Wilcoxon test.

The BCRs were also analyzed in the bulk RNA-seq data using the standard workflow of TRUST4. And the results showed that the frequency of clonotypes in JIA was significantly increased (*p* < 0.05; Figure 2A). Additional analyses were performed to examine the distribution of BCR clonotypes frequencies, uncovering categories of small, medium, large, and hyperexpanded frequencies. The clonotype distributions at small frequencies were significantly increased in JIA (*p* < 0.05), whereas the distributions at medium and large frequencies and hyperexpanded frequencies were increased in HC(*p* < 0.05; Figure 2B). The results suggested that the JIA exhibited a greater degree of dominant clonal expansion and a lower BCR repertoire diversity. The results of distributions of CDR3 amino acid sequence length showed that the most common CDR3 amino acid sequences for both JIA and HC are 16. CDR3 amino acid sequence length did not significantly differ between the JIA and HC groups (Figure 2C). Both the Chao1 and InvSimpson indices were utilized to evaluate the diversity of the CDR3 amino acid sequences. Compared to HC, the Chao1 and InvSimpson indices were significantly increased in JIA (*p* < 0.05; Figure 2D). To measure the frequency of TRBJ and TRBV genes among the JIA and HC groups, we generated a bar chart depicting the common usage frequencies of these genes (Figures 2E,F). The results showed that there was no difference between these two groups.

#### 3.1.2 ScRNA-seq analysis of PBMCs

**FIGURE 3:**
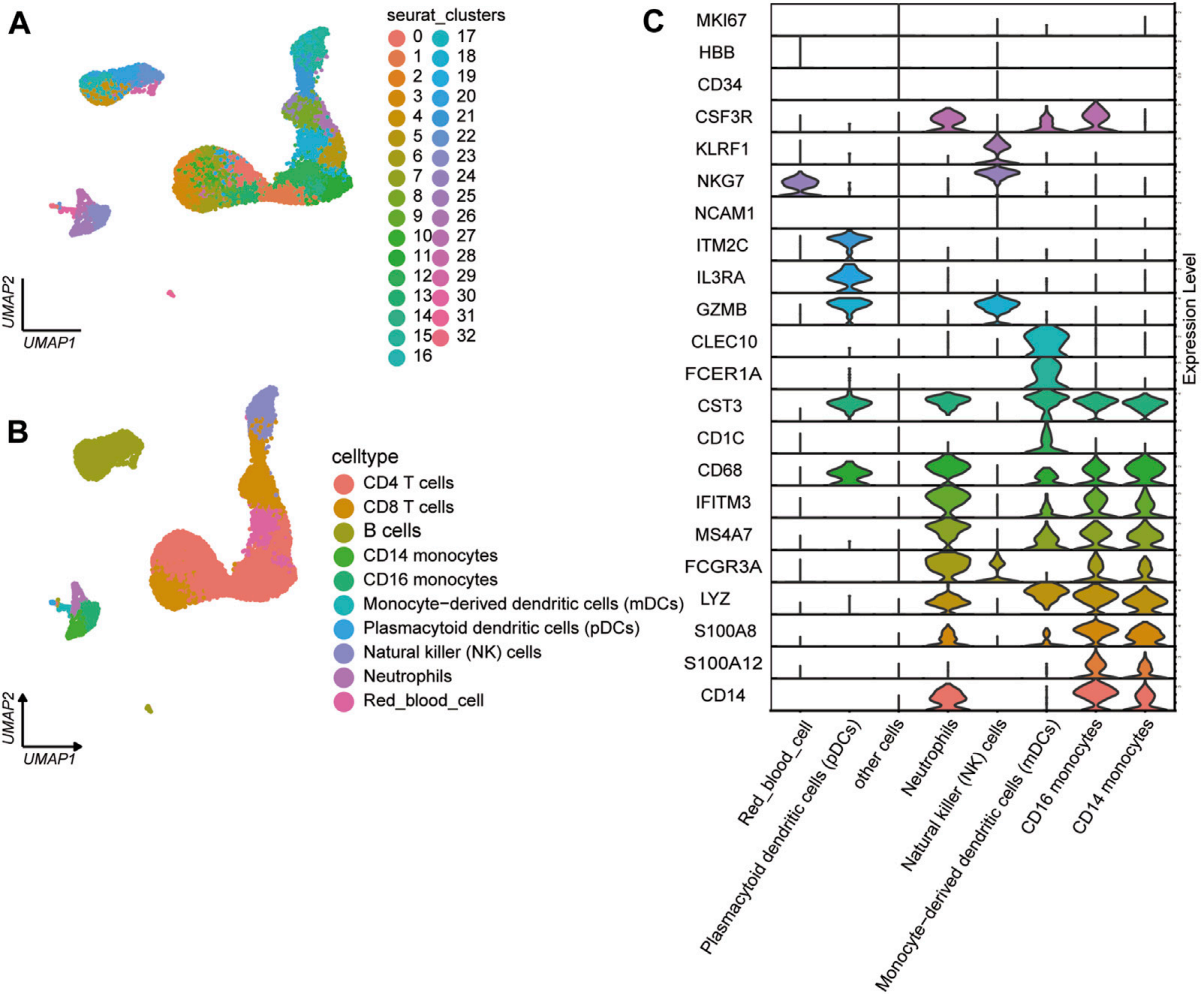
Single-cell transcriptome profiles of PBMCs from the JIA and HC groups. **(A)** Identification of cell populations. A total of 8 PBMC samples from the HC group (n = 2) and JIA group (n = 6) were sequenced, and a total of 23,104 high-quality single cells were obtained. After quality control, 20,986 cells were obtained, and 33 clusters of cells were identified via UMAP. Each dot corresponds to a single cell and is colored according to the cell type. Each color represents a cluster. **(B)** UMAP charts of 20,986 single cells colored according to cell type. The 33 cell clusters were further identified as 10 cell types. UMAP was used to identify and visualize these celltypes. Each dot represents an individual cell and is colored according to its corresponding cell type. **(C)** Canonical cell markers were utilized to assign cell identities to the clusters represented in the UMAP plot.

We analyzed the single-cell transcriptional profiles of PBMCs from JIA patients by examining scRNA-seq data from 2 healthy individuals and 6 individuals with JIA. A total of 23,104 high-quality single cells were obtained from 8 samples, with 20,986 cells used for cell type identification after filtering. Among these, 4,520 cells belonged to the HC group, and 16,466 cells belonged to the JIA group. The filtered data underwent integration, dimensionality reduction, and clustering using an unsupervised method, resulting in the identification of 33 major cell types visualized through UMAP (Figure 3A). Ten clusters were distinguished based on the expression of characteristic gene markers, representing cell types such as CD14 monocytes, CD16 monocytes, CD4 T cells, CD8 T cells, B cells, monocyte-derived dendritic cells (mDCs), plasmacytoid dendritic cells (pDCs), natural killer (NK) cells, neutrophils, and red blood cells (Figure 3B). The typical marker genes for each cell type are depicted in Figure 3C.

#### 3.1.3 Phenotypic characteristics of cell types in JIA

**FIGURE 4:**
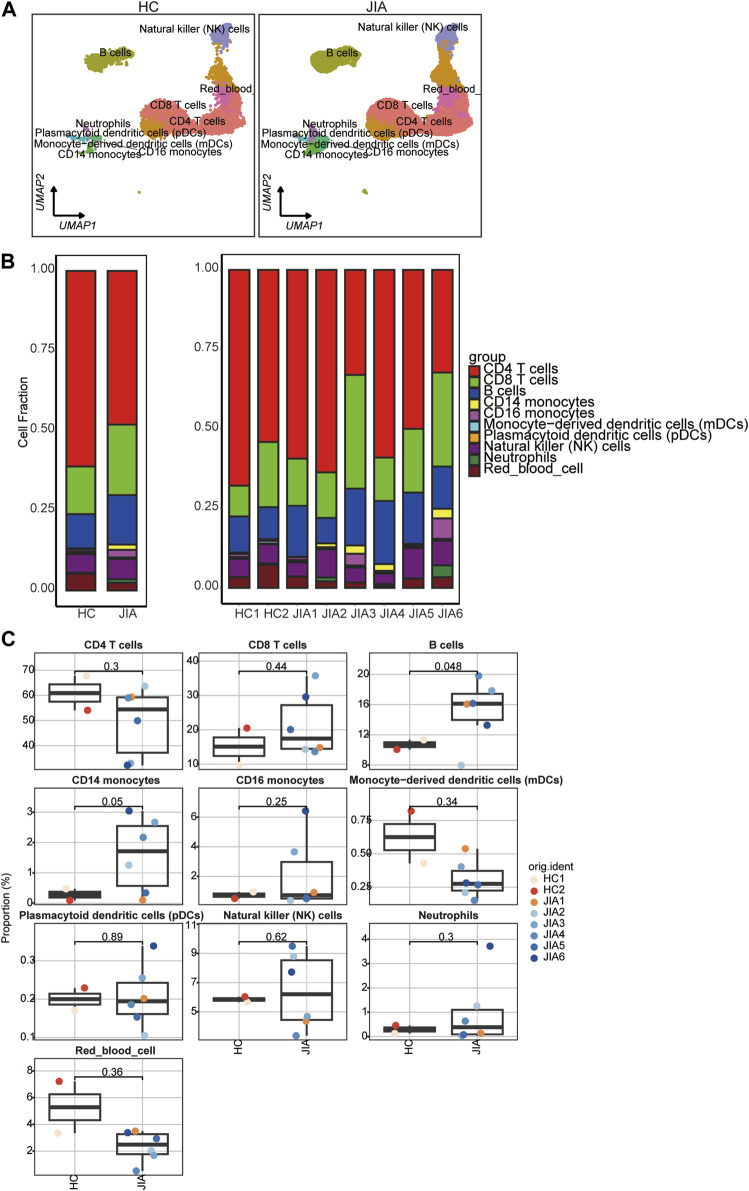
Differences in cell composition between the JIA and HC groups. **(A)** UMAP plots of the HCs and JIA patients. Each dot corresponds to a single cell and is colored according to the cell type. **(B)** The average proportion of each cell type was derived from the HCs and JIA groups. The left picture depicts the average proportion of each cell type derived from the two groups. The calculation method was as follows: (the number of specific cell clusters in one group) ⁄ (the number of total cells in one group). The dot plot in the upper panel of the right picture shows the sum of the absolute counts of cell subsets in the PBMCs of each sample, and the bottom bar plot shows the cell compositions at the single sample level. The calculation method was as follows: (the number of specific cell clusters in one sample) ⁄ (the number of total cells in one sample). **(C)** Box charts showing the proportion of each cell type among the total PBMCs in each sample across the three groups (n = 2 in the HC group, n = 6 in the JIA group). *p* < 0.05 was considered to indicate statistical significance.

To illustrate the differences in cellular composition, the expression profiles of 10 cell types within these two groups are depicted in Figure 4A. The relative percentages of all cell types among PBMCs from each individual were calculated (Figures 4B,C). The results showed that the relative percentages of CD14 monocytes were significantly increased in the JIA group.

#### 3.1.4 Characterization of T cells between the JIA and HC groups

**FIGURE 5:**
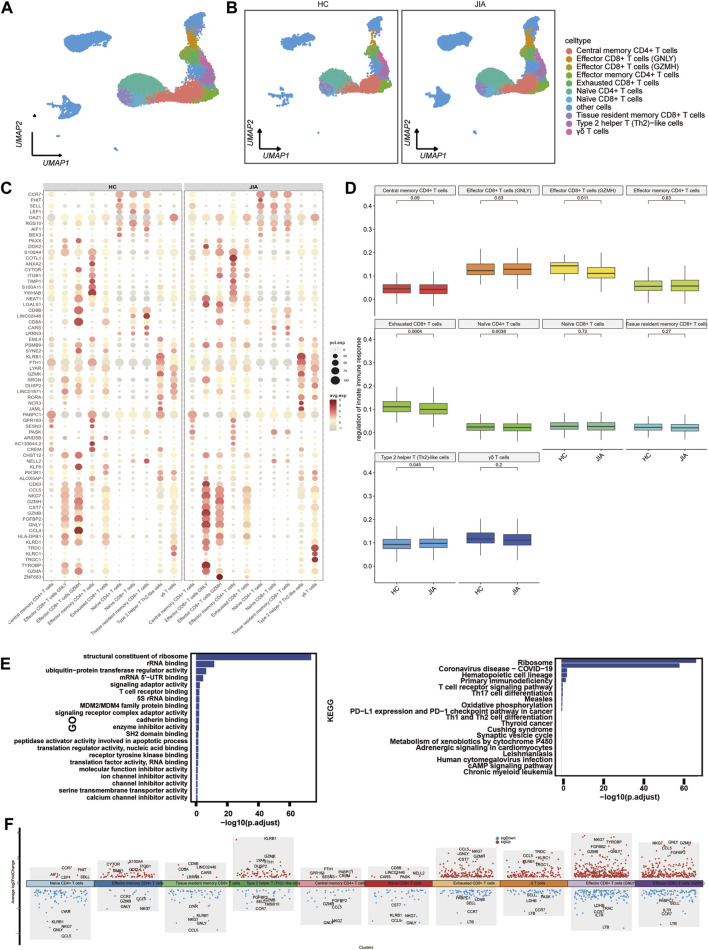
Characterization of T cell subsets between the JIA and HC groups. **(A)** UMAP visualization of distinct populations of T-cell subsets is depicted. **(B)** UMAP plots of T-cell subsets acquired from the HCs and the JIA group are shown. **(C)** A dot plot is presented, illustrating the gene expression and percentage of T cells expressing the top 70 genes that exhibited differential expression. **(D)** The size of each dot corresponds to the percentage of cells expressed, while the color represents the expression level on a logarithmic scale. Statistical analysis was conducted using the Wilcoxon rank sum test. **(E)** Enrichment analyses of the differentially expressed genes (DEGs) were performed using the Biological Process (BP) database within the Gene Ontology (GO). The GO terms are annotated with their respective names and IDs and are arranged in descending order based on the logarithm of the reciprocal of the *p*-value (-log10). The top 20 enriched GO terms are displayed. Enrichment analyses of the Kyoto Encyclopedia of Genes and Genomes (KEGG) were performed. The top 20 enriched GO terms are displayed. **(F)** Scatter plot showing changes in DEGs, where red represents upregulation and blue represents downregulation.

We examined the changes in T cells post JIA onset by analyzing T cells within PBMCs. UMAP visualization of canonical T-cell markers revealed 10 distinct T-cell subpopulations (Figure 5A), including effector CD8^+^ T cells (GNLY), effector CD8^+^ T cells (GZMH), central memory CD4^+^ T cells, effector memory CD4^+^ T cells, naïve CD8^+^ T cells, naïve CD4^+^ T cells, exhausted CD8^+^ T cells, tissue resident memory CD8^+^ T cells, type 2 helper T (Th2)-like cells, γδ T cells and other cells (Figure 5B). Functional enrichment analysis using gene ontology (GO) highlighted the significant involvement of T cells, such as structural constituent of ribosome, rRNA binding, ubiquitin-protein transferase regulator activity, mRNA 5′-UTR binding, and signaling adaptor activity. The results of KEGG pathway indicated T cells participation in pathways like the ribosome, coronavirus disease-COVID-19, primary immunodeficiency, hematopoietic cell lineage, T-cell receptor signaling pathway, and Th17 cell differentiation pathways (Figure 5E). The expression profiles of differentially expressed genes (DEGs) in T cells were depicted visually, with red representing upregulation and blue representing downregulation. Figure 5F highlights the top five genes displaying the most notable changes.

#### 3.1.5 Characterization of B cells between the JIA and HC groups

**FIGURE 6:**
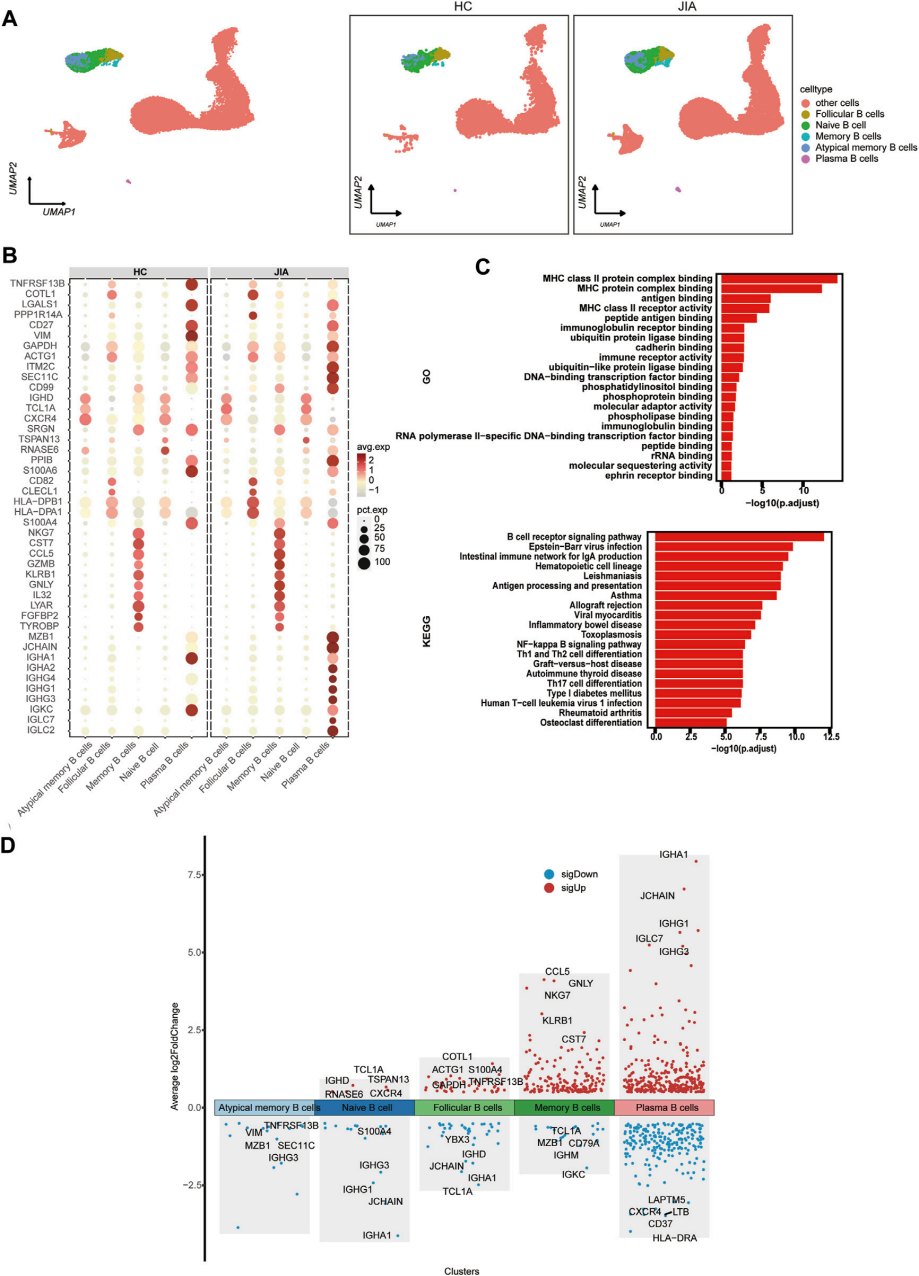
Characterization of B cell subsets between the JIA and HC groups. **(A)** UMAP plots of distinct populations of memory B cells, naïve B cells, activated B cells and plasma B cells are shown. Additional cell clusters are denoted as other unidentified cells. UMAP plots of B-cell subsets acquired from the HCs and from the JIA group are shown. **(B)** A dot plot is presented, illustrating the gene expression and percentage of B cells expressing the top 50 genes that exhibited differential expression. **(C)** Enrichment analyses of the DEGs were performed using the BP database within the GO database. The GO terms are annotated with their respective names and IDs and are arranged in descending order based on the logarithm of the reciprocal of the *p*-value (-log10). The top 20 enriched GO terms are displayed. KEGG enrichment analyses were performed. The top 20 enriched GO terms are displayed. **(D)** Scatter plot showing changes in DEGs, where red represents upregulation and blue represents downregulation.

We investigated the changes in B cells post JIA onset through a detailed analysis of B cells within PBMCs. By leveraging canonical B-cell markers, UMAP visualization identified 5 distinct B-cell subpopulations, including follicular B cells, naïve B cells, atypical memory B cells, and plasma B cells (Figure 6A). Functional enrichment analysis using GO highlighted the significant role of B cells, such as MHC class II protein complex binding, MHC protein complex binding, antigen binding, MHC class II receptor activity, and peptide antigen binding. Furthermore, KEGG pathway analysis indicated that B cells’ involvement in pathways like the B-cell receptor signaling pathway, Epstein–Barr virus infection pathway, intestinal immune network for IgA production, hematopoietic cell lineage pathway, and leishmaniasis pathway (Figure 6C). The expression profile of DEGs in B cells was visualized. The figure highlights the five genes showing the most significant alterations (Figure 6D).

#### 3.1.6 HdWGCNA analysis of T cells

**FIGURE 7:**
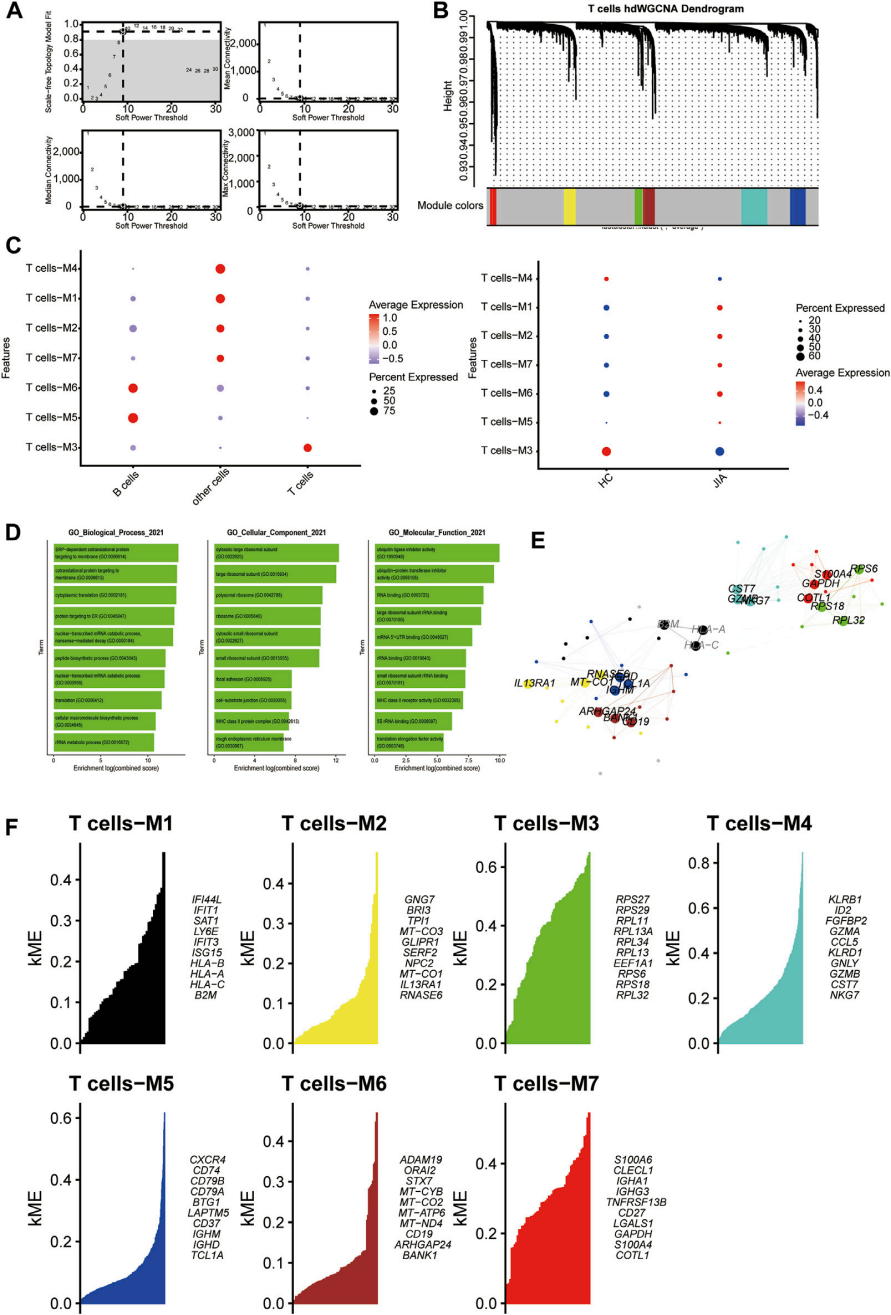
Identification of gene coexpression modules among T cells. **(A)** Weigned gene coexpression network analysis was performed with T cells. **(B)** A Weighed gene coexpression network analysis was performed on T cells. **(C)** Dot plot for enrichment of modules in different cell types and different groups. **(D)** Dot plot of the GO functional enrichment analysis of the T cells-M3 module. **(E)** Protein–protein interaction network demonstrating the interactions within Module T cells-M3 **(F)**. The top ten genes of each module calculated according to connectivity.

With a soft threshold of 9, we built an unscaled network for T cells, resulting in the discovery of seven gene modules (Figures 7A,B). The T cell-M3 module showed significant enrichment in T cells (Figure 7C). Subsequently, we performed GO enrichment analyses for genes within the T cell-M3 module, highlighting their functional roles in biological process, cellular component, and molecular function (Figure 7D). Furthermore, we established a PPI network to validate the interactions among hub genes within the T cell-M3 module (Figure 7E). Lastly, we explored gene expression modules in T cells and identified key hub genes (Figure 7F).

#### 3.1.7 HdWGCNA analysis of B cells

**FIGURE 8:**
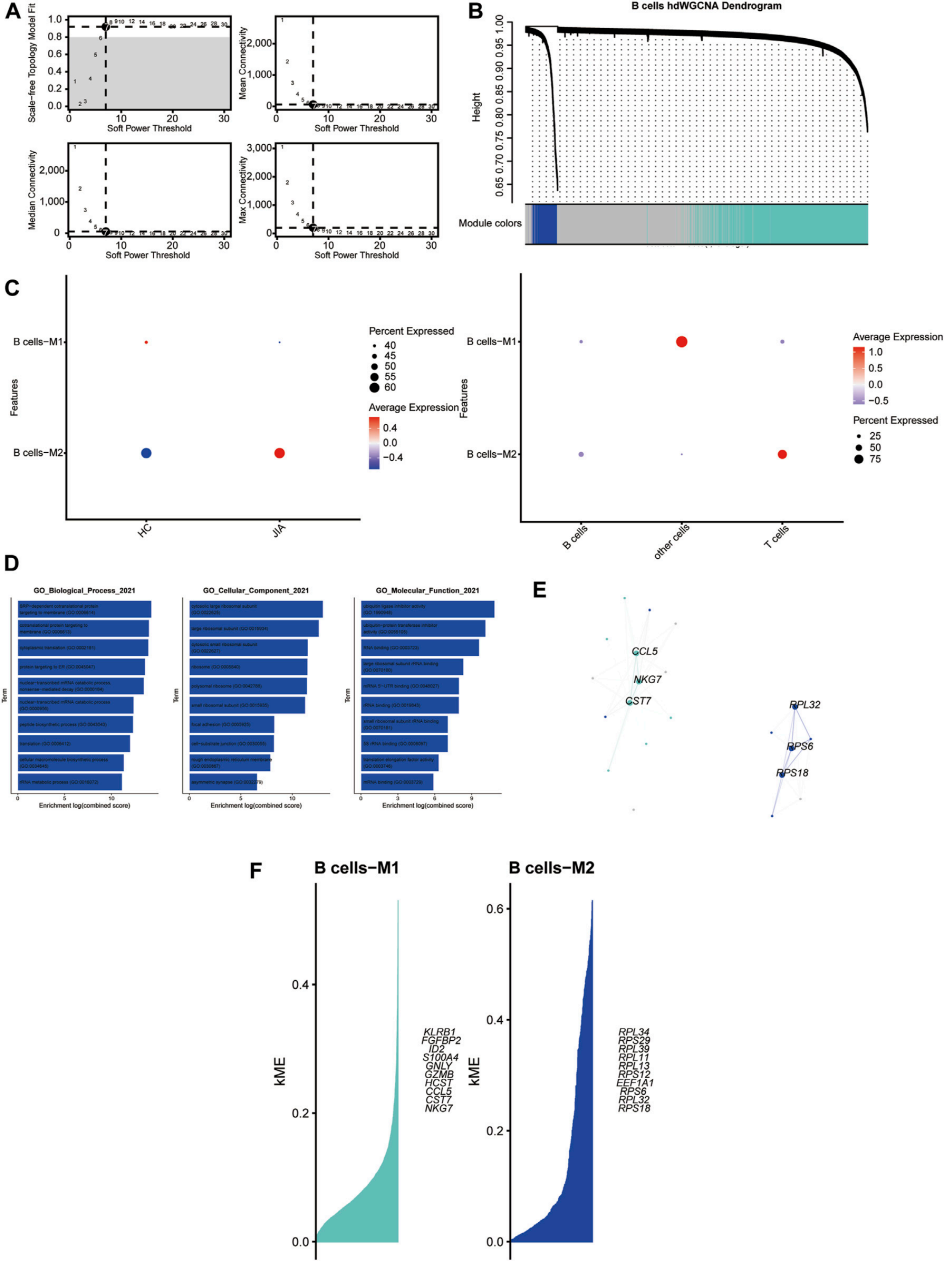
Identification of gene coexpression modules among B cells **(A)** Weigned gene coexpression network analysis was performed with B cells. **(B)** A weighted gene coexpression network analysis was constructed among the B cells. **(C)** Dot plot for enrichment of modules in different cell types and different groups. **(D)** Dot plot of the GO functional enrichment analysis of module B cells-M2. **(E)** Protein-protein interaction network demonstrating the interactions within Module B cells-M2 **(F)**. The top ten genes of each module calculated according to connectivity.

With a soft threshold of 7, we built an unscaled network for B cells, resulting in the discovery of seven gene modules (Figures 8A,B). The B cells-M2 module showed significant enrichment in B cells (Figure 8C). Subsequently, we performed GO enrichment analyses for genes within the B-cell-M2 module, highlighting their functional roles in biological process, cellular component and molecular function (Figure 8D). Furthermore, we established a PPI network to validate the interactions among hub genes within the B-cell-M2 module (Figure 8E). Lastly, we explored gene expression modules in B cells and identified key hub genes (Figure 8F).

#### 3.1.8 Analysis of cell-cell interactions related to T cells

**FIGURE 9:**
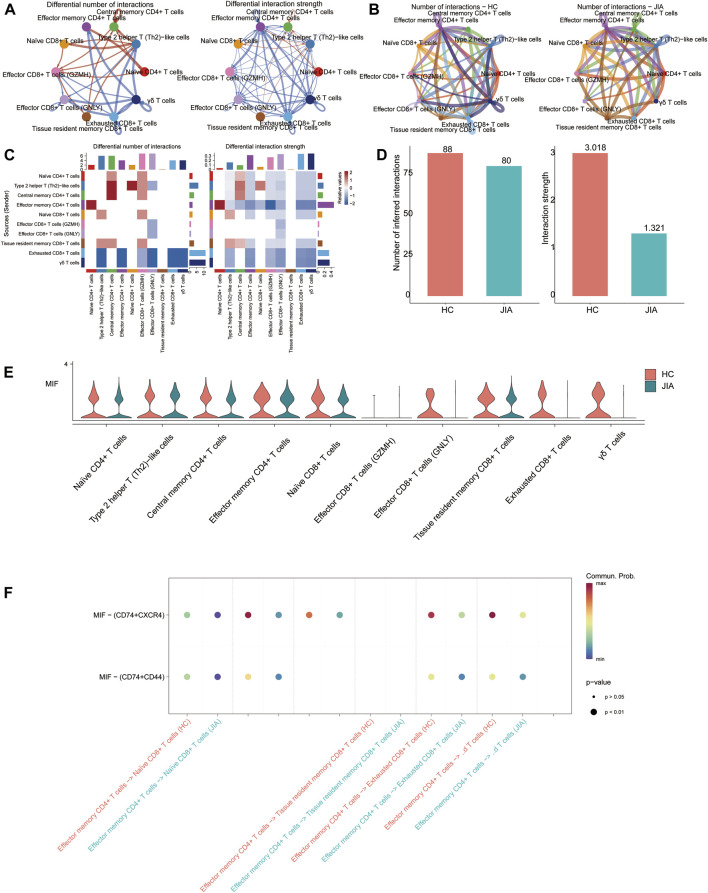
CellChat analysis of the interactions between T-cell subsets. **(A, B)** Circle plots illustrate the number and strength of interactions between T-cell subsets in the HC and JIA groups. **(C, D)** Identification of signaling pathways in cells *via* network centraility analysis. **(E)** The gene targets from the MIF signaling and the expression levels among HC and JIA **(F)** Discovery of dominant cell communication patterns. The inferred outgoing communication patterns of secreting cells, which show the correspondence between the inferred latent patterns and cell groups, as well as signaling pathways. The thickness of the flow indicates the contribution of the cell group or signaling pathway to each latent pattern.

Cell-cell communication results showed that the number of communications for T-cell subgroups in JIA patients was 80, with a communication strength of 1.321; however, in HC, the number of communications was 88, with a communication strength of 3.018. And the results indicated that the significant role of the MIF signaling pathway in JIA. The MIF pathway contains two types of ligand-receptor pairs: MIF-(CD74+CXCR4) and MIF-(CD74^+^CD44). According to the calculations based on gene expression, MIF-(CD74+CXCR4) had a greater contribution to intercellular communication (Figure 9).

#### 3.1.9 Trajectory analysis related to T and B cells

**FIGURE 10:**
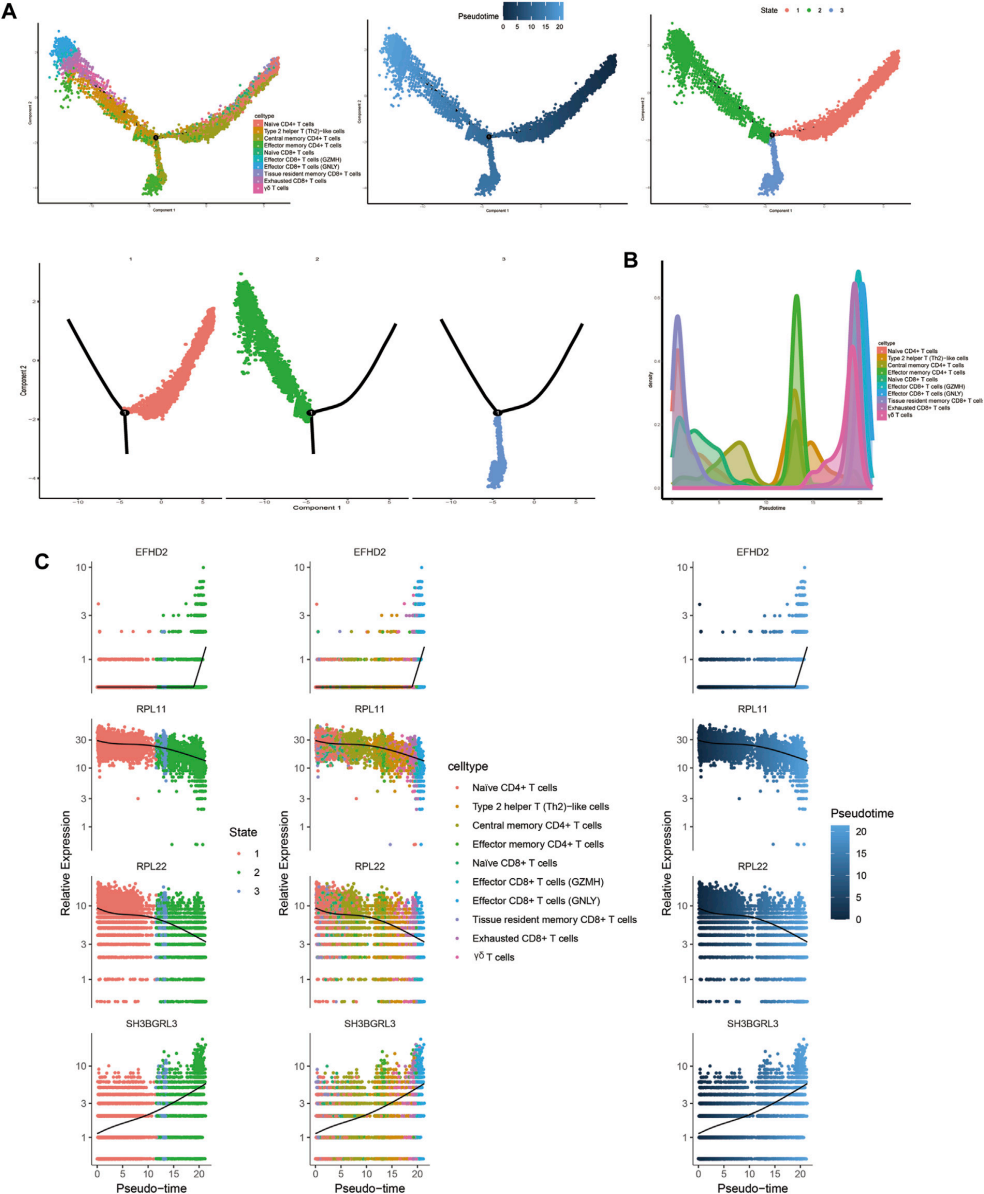
Characterization of the landscape of T cells and developmental trajectories of T cells in JIA. **(A)** Developmental trajectories of the T-cell lineage inferred using monocle2; each cell subtype is marked with a different color. **(B)** Cell density variation in T-cell subtypes during pseudotime (top). **(C)** Pseudoscatter plots showing the expression variation and distribution of some specific genes during pseudotime, color coded by cell type.

**FIGURE 11:**
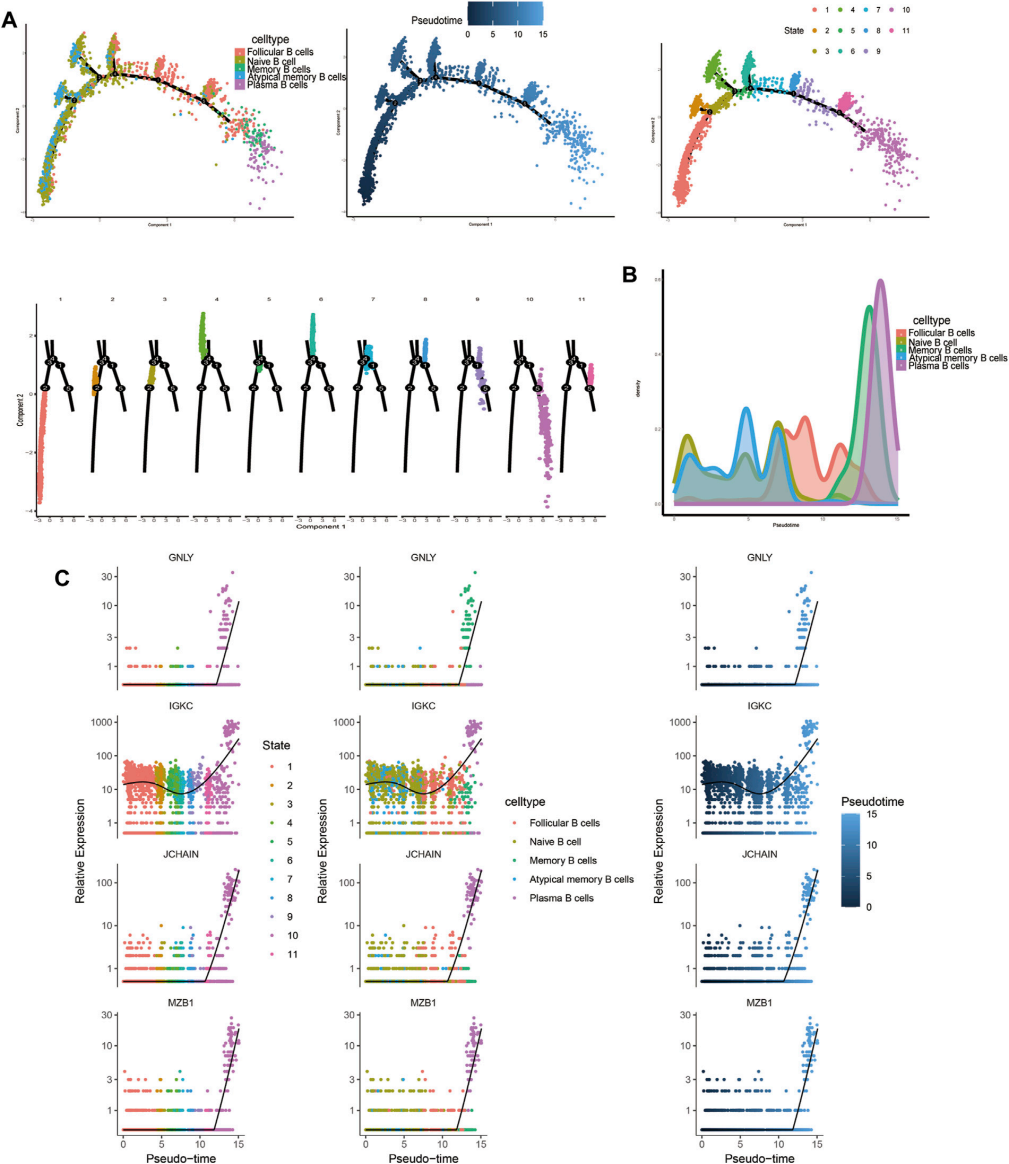
Characterization of the landscape of B cells and developmental trajectories of B cells in JIA. **(A)** Developmental trajectories of the B-cell lineage inferred using monocle2; each cell subtype is marked with a different color. **(B)** Cell density variation in B-cell subtypes during pseudotime (top). **(C)** Pseudoscatter plots showing the expression variation and distribution of some specific genes during pseudotime, color-coded by cell type.

Trajectory analysis results revealed that T-cell subsets could be categorized into 3 differentiation states, with naïve CD4^+^ T cells, central memory CD4^+^ T cells, and naïve CD8^+^ T cells representing early developmental stages, while effector CD8^+^ T cells (GNLY), effector CD8^+^ T cells (GZMH), and exhausted CD8^+^ T cells were identified as being in the terminal stage of development (Figure 10). Similarly, for B-cell subsets, the analysis indicated 5 clusters corresponding to 5 differentiation states, with naïve B cells and atypical memory B cells in the early developmental stage, and plasma B cells and memory B cells in the terminal stage of development (Figure 11).

#### 3.1.10 Transcription factor regulatory network of T and B cells

**FIGURE 12:**
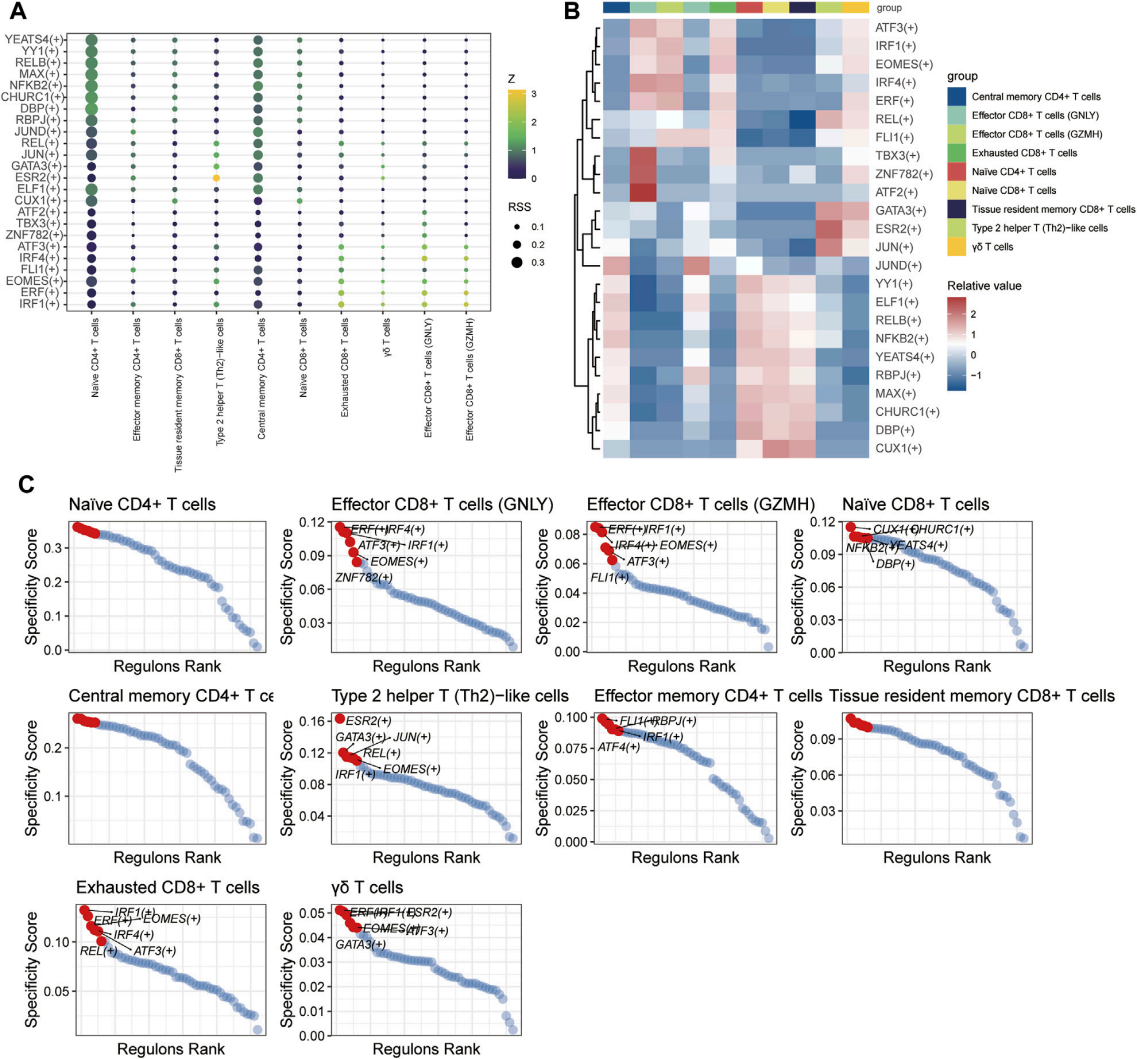
The TF of T-cell subgroups predicted by SCENIC analysis. **(A)** Dimplot of the main TFs in the T-cell subgroups. **(B)** Heatmap of the expression levels of selected TFs in T-cell subgroups. **(C)** RANK plot of T-cell subgroup TFs.

**FIGURE 13:**
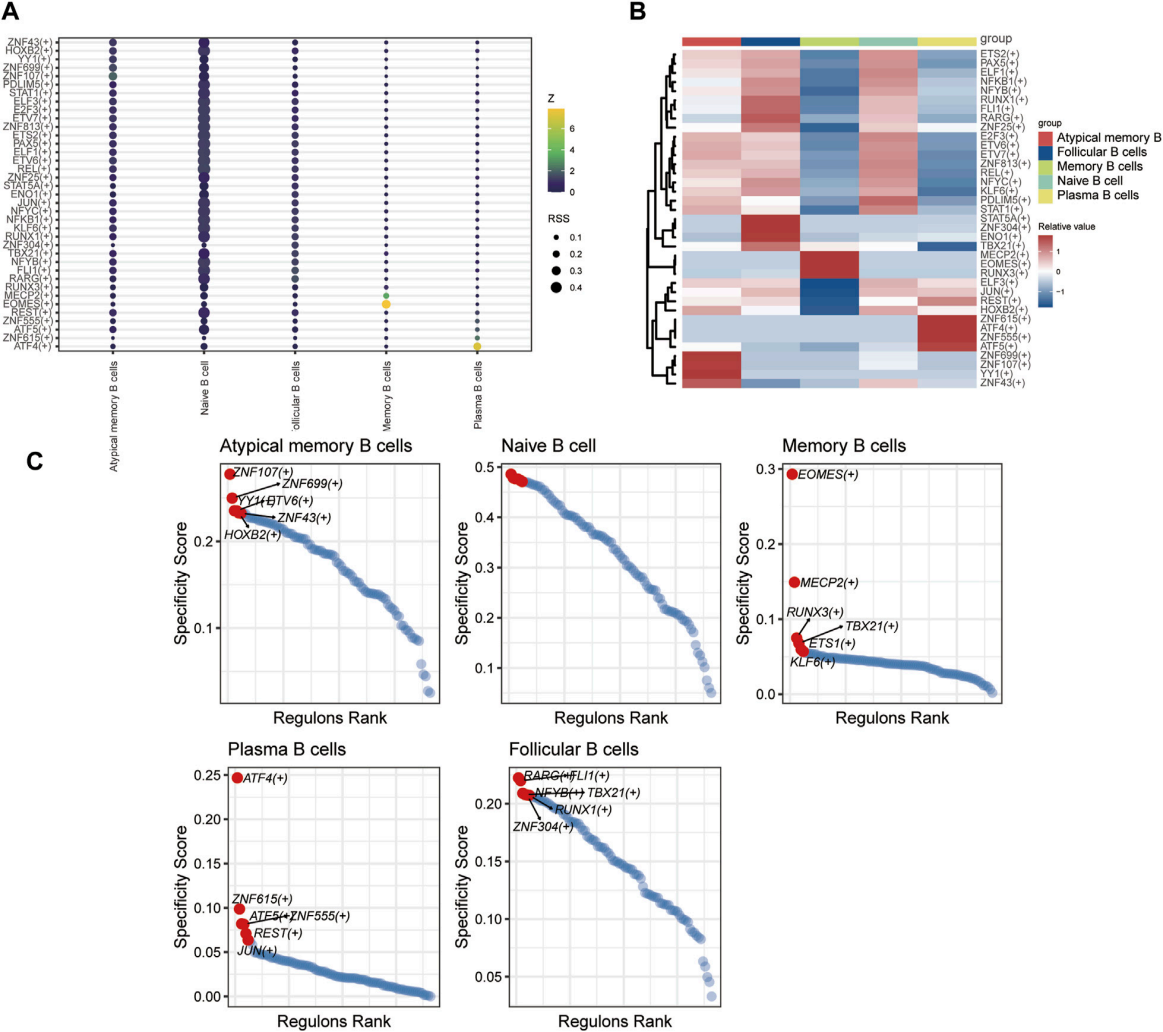
The TF of B-cell subgroups predicted by SCENIC analysis. **(A)** Dimplot of B-cell subgroup main TFs. **(B)** Heatmap of the expression levels of selected TFs in B-cell subgroups. **(C)** RANK plot of B-cell subgroup TFs.

SCENIC analysis was utilized to predict transcription factors in both T and B cells, and the results were visualized using R. In T cells, the analysis revealed high expression of transcription factors in naïve CD4^+^ T cells and central memory CD4^+^ T cells (Figure 12). Similarly, in B cells, the analysis indicated that transcription factors were highly expressed in naïve B cells (Figure 13).

## 4 Discussion

Although the analysis of immune cells in peripheral blood can provide valuable insights into the coordinated immune response during pathogen infections, the current understanding of the underlying mechanisms of JIA onset and progression and its association with inflammation is limited. To address this issue, we conducted a comprehensive investigation of the immune repertoire (TCR, BCR) and scRNA-seq data. Our objective was to analyze transcriptome sequencing data and uncover the properties and molecular regulatory mechanisms underlying JIA.

Our study has illustrated several important findings related to JIA gene expression heterogeneity. Firstly, the JIA group had significantly greater clonotype distributions at small frequencies. The clonotype distributions at small frequencies were significantly greater in the JIA group, whereas the distributions at medium and large frequencies and hyperexpanded frequencies were greater in the HC group. These results suggest that the JIA group has lower TCR and BCR repertoire diversity and a greater degree of dominant clonal expansion, indicating a more specific T-cell response against JIA. Secondly, the JIA group exhibited significantly greater Chao1 and InvSimpson indices. These findings provide valuable insights into the features of the TCR and BCR repertoires and suggest an immune response to common autoantigens in JIA patients, which corresponds to our hypothesis that autoimmune diseases could lead to the increase in specific CDR3 amino acid sequences. Moreover, these findings could aid in the development of targeted biotherapy and the diagnosis of JIA patients.

Compared to HC group, the relative percentages of CD14 monocytes in the JIA group were significantly increased in scRNA-seq data. Monocytes are vital cells of the innate immune system that circulate within the body (Yona and Jung, 2010). The increase in monocytes may serve as an indicator to observe the progression of JIA disease. We also conducted HdWGCNA to investigate the characteristics of the gene coexpression regulatory modules involved in JIA pathogenesis. The results revealed significant enrichment of the T cell-M3 and B cell-M2 modules in the JIA group. CellChat is a tool that is able to quantitatively infer and analyze intercellular communication networks from scRNA-seq data (Jin et al., 2021). Our findings revealed the significant role of the macrophage inhibitory factor (MIF) signaling pathway in JIA, indicating the vital role of the MIF pathway in the maintenance of immune tolerance and inflammation. MIF is a cytokine expressed in a diverse range of cell types, including hematopoietic, epithelial, endothelial, mesenchymal, and neuronal cells (Bilsborrow et al., 2019; Luo et al., 2021; Sumaiya et al., 2022). Altered MIF expression has been implicated in numerous diseases, ranging from inflammatory disorders such as JIA, lupus, and rheumatoid arthritis to organ pathologies such as heart failure, myocardial infarction, acute kidney injury, organ fibrosis, and various malignancies.

To further elucidate the developmental stages of T/B-cell subsets, we employed Monocle2 software to conduct pseudotime series analysis (Tao et al., 2023). T/B-cell plasticity refers to the capacity of differentiated T/B cells to polarize to other phenotypes in response to a changed microenvironment or context and obtain characteristics of other subsets. The outcomes of our study revealed varying levels of differentiation in PBMCs from both T and B cells, suggesting that targeting their differentiation could be a potential approach for treating JIA. To gain insight into the transcriptional regulation of JIA, we employed SCENIC analysis to predict the involvement of transcription factors (Aibar et al., 2017; Van de Sande et al., 2020). The results showed that transcription factors were highly expressed in naïve CD4^+^ T cells, central memory CD4^+^ T cells, and naïve B cells. Notably, the transcriptional activity of these 3 cell groups may surpass that of other cell groups.

In conclusion, we examined the immune repertoire and the mechanisms underlying the onset and progression of JIA. These findings indicate that there are significant differences in the TCR and BCR repertoires between the JIA and HC, with specific genes identified that could enhance our understanding of JIA. Additionally, scRNA-seq data analysis revealed an increase in CD14 monocytes in JIA, and the involvement of MIF signaling pathways was highlighted through cell-cell communication analysis. Our findings revealed the changes and regulatory processes occurring in PBMCs during the development and progression of this disease. This study provides valuable insights into potential factors that may contribute to investigating the specific role of these pathways and cell population.

## Data Availability

The original contributions presented in the study are included in the article/Supplementary material, further inquiries can be directed to the corresponding author.
